# The deubiquitinating enzyme USP1 modulates ERα and modulates breast cancer progression

**DOI:** 10.7150/jca.50477

**Published:** 2020-10-12

**Authors:** Zhiguo Niu, Xin Li, Suyin Feng, Qingsong Huang, Ting Zhuang, Cheng Yan, Hui Qian, Yinlu Ding, Jian Zhu, Wenrong Xu

**Affiliations:** 1 Jiangsu Key Laboratory of Medical Science and Laboratory Medicine, School of Medicine, Jiangsu University, Zhenjiang, Jiangsu, 212000, China.; 2 Henan Key Laboratory of Immunology and Targeted Drugs, School of Laboratory Medicine, Xinxiang Medical University, 453000, China.; 3 Department of Neurosurgery, Affiliated Hospital of Jiangnan University, 200 Huihe Road, Wuxi, 214000, Jiangsu, China.; 4 School of Medicine, Xinxiang University, Xinxiang, 453003 Henan P.R. China.; 5 Department of general surgery, the Second Hospital, Cheeloo College of Medicine, Shandong University, Jinan, China, 250033.

**Keywords:** USP1, ERα, Breast cancer, Deubiquitin, Stabilize

## Abstract

Breast cancer is one of the most common malignancies worldwide, while the luminal types (ERα positive) accounts for two third of all breast cancer cases. Although ERα positive breast cancer could be effective controlled by endocrine therapy, most of the patients will develop endocrine resistance, which becomes a headache clinical issue for breast cancer field. Endocrine resistance could be caused by multiple pathway disorders, the dys-regulation of ERα signaling might be a critical factor, which makes it urgent and important to reveal the potential molecular mechanism of ERα signaling. In our current study, we identified a new deubiquitination enzyme USP1 through screening the whole DUB (Deubiquitinases) siRNA library. The expression of USP1 is elevated in human breast cancer compared with normal mammary tissues. Importantly, USP1 expression levels are specially correlated with poor survival in ERα positive patients. USP1 depletion inhibited breast cancer cell progression and ERα signaling activity. Immuno-precipitation assays indicate that USP1 associates with ERα and promotes its stability possibly via inhibiting ERα K48-linked poly-ubiquitination. In conclusion, our data implicate a non-genomic mechanism by USP1 via stabilizing ERα protein controls ERα target gene expression linked to breast cancer progression.

## Introduction

Breast cancer is one of the most common women cancers worldwide, while ERα positive breast cancer is the major subtype of breast malignancy 1. Compared with ERα negative breast cancer, ERα positive subtype could benefit from endocrine therapy and has better overall survival 2. However, the occurrence of endocrine resistance becomes a major challenge in ERα positive breast cancer for both clinics and basic researches 3, 4. The further understanding of ERα signaling activity, including the ERα expression and stability, is critical in developing novel therapeutics for breast cancer.

ERα signaling was recognized as the major driver for breast cancer for more than 30 years 5. Estrogen, which binds to ERα protein, has a critical role in mammary epithelial cell development and breast cancer proliferation via regulating cell cycle-related genes 6. Estrogen-stimulated cell proliferation is activated through binding to ERα in the ligand-dependent manner. When ERα is activated, it endures the conformational change, trans-locates into the nuclear and promotes the ERα target gene expression through binding to their promoter regions. Several studies confirmed that breast cancer showed elevated ERα mRNA level due to dys-regulation of ERα by transcriptional factors or co-activators, which bind to ESR1 promoter regions 7, 8. However, recently studies showed that the post-translational modifications, such as ubiquitination, sumoylation and phosphorylation, are also play important roles in regulating ERα protein stability or activity 9-11. More importantly, the ubiquitin proteasome system has a center role in regulating ERα protein level, while several E3 ubiquitin ligases demonstrated to regulate ERα stability, including RNF31, TRIM56 and MDM2 9, 12. However, the ubiquitination process could be counteracted by deubiquitination. The DUBs (Deubiquitinases) function to cleave the ubiquitin chains from the substrate proteins and modulate protein stability or activity in several biological processes 13.

Despite the importance of DUBs in protein regulation, still little is known about DUBs function in regulation estrogen signaling in breast cancer. We did the screening of human DUBs for key regulators of ERα by DUBs siRNA library. Ubiquitin specific protease 1 (USP1) was observed to play critical role in ERα signaling in breast cancer. Previous studies showed that USP1 was located in the nuclear, while it regulated cell cycle progression and DNA damage response. Several oncological studies showed that USP1 was elevated in a few human cancers and mediated chemotherapy resistance 14, 15. In our current study, we reported USP1 associated with ERα, inhibited ERα poly-ubiquitination and degradation in breast cancer cells, which indicated that USP1 linked to breast cancer proliferation and invasion via estrogen signaling.

## Materials and Methods

### RNA extraction and qPCR analysis

Total RNA was used to extract by RNeasy plus mini kits according to the protocol (Tiangen). Real-time PCR was showed as previously described 16. 36B4 was used for internal reference. The primer sequences were displayed here. USP1: F: CTC CCG GGA TGT AGT TGG TG; R: ATT ATA TCT GGT CAT GGC CCA AAG. 36B4: F: ggc gac ctg gaa gtc caa ct; R: cca tca gca cca cag cct tc. GREB1 F: CGT GTG GTG ACT GGA GTA GC, R: ACC TCT TCA AAG CGT GTC GT. ER F: GCT ACG AAG TGG GAA TGA TGA AAG, R: TCT GGC GCT TGT GTT TCA AC. PS2 (TFF1) F: TGG GCT TCA TGA GCT CCT TC, R: TTC ATA GTG AGA GAT GGC CGG.

### Cell culture

We acquired the MCF-7, T47D and HEK293 cells orm American Type Culture Collection (ATCC). T47D cells were maintained with RPMI-1640 (42401, Life Technologies) supplemented with 2 mM L-glutamine (25030, Life Technologies) and 10% FBS. MCF-7 and HEK293 were grown with Dulbecco's Modified Eagle's Medium that contains 4,5 g/L glucose and 4 mM L-glutamine (DMEM, 41965, Life Technologies) supplemented with 10% Fetal Bovine Serum (FBS, 10270, Life Technologies). All cell lines are characterized by cell line authentication. The cell line authentication via Short Tandem Repeat (STR) is performed via PowerPlex 21 system. The STR data of MCF-7 and T47D cell lines are found consistent with STR data in ATCC.

### Plasmids and siRNA

The Flag-USP1 plasmid was acquired from Origene. The HA-K48 and Ub wild type plasmids were acquired from our previous study 9. The ESR1 plasmid was acquired from previous studies 17. The Lipofectamin 2000 (1662298, Invitrogen) was used for the plasmids transfection. Small interfering RNAs were used for specific gene knocking-down. The USP1 siRNA sequences were: GUAUACUUCAGGUAUUAUAdTdT; UAUAAUACCUGAAGUAUACdTdT and CCAUACAAACAUUGGUAAAdTdT; UUUACCAAUGUUUGUAUGGdTdT. The negative control siRNA sequences were: UUCUCCGAACGUGUCACGUTT; ACGUGACACGUUCGGAGAATT. The RNAiMAX reagent (13778150, invitrogen) was used for siRNA transfection.

### Cell proliferation assay

MCF-7 cells were transfected with siUSP1 or siControl into 24-well plates. Twenty-Four hours after transfection, the cells number was countered and 4000 cells were seeded into 96-well plates. The relative cell viability was measured at indicated time points. Cell numbers were determined using the WST-1 cell proliferation reagent as previously described.

### EdU staining assay

For ethynly-deoxyuridine (EdU) labeled DNA, cells were incubated with EdU for 2 hours. Later on, the cells were fixed in cell culture plates with 4% formalin. The EdU positive cells were counted with statistical analysis.

### Wound healing assay

Fifty nM USP1 siRNA or siControl were transfected into MCF-7 cells. After twenty-four hours, cells were seeded into 12-well paltes with 1%FBS. The cells were 100% confluence. The yellow pipette tips were applied for straight scratch. The wound distance was measured at indicated time points and normalized with starting time point. The wound healing recovery was expressed as: [1-(Width of the wound at a given time/width of the wound at t=0)] ×100%.

### Western blotting

Cells were harvested and lysed with RIPA buffer. Proteins were separated by electrophoresis on SDS-polyacrylamide gel electrophoresis (PAGE) and electro-transferred to PVDF membrane. The antibodies used in this study were listed here: Anti- ERα (D8H8, 8644, Cell signaling Technology); Anti- ERα (SC-56833, Santa Cruz); Anti-USP1 (A301-699A, Thermo Fisher Scientific); Anti-HA (MMS-101R, COVANCE); Anti-myc (9E10, ab32, Abcam); Anti-myc (Ab9106, Abcam); Anti-Flag (Ab49763, Abcam); Anti-GFP (Ab290, Abcam). Membranes were then washed with PBS for three times and incubated with secondary antibodies Peroxidase-Conjugated AffiniPure Goat Anti-Mouse IgG or Goat Anti-Rabbit IgG. Fluorescent signals were visualized with ECL system. (amersham imager 600, USA).

### Luciferase assay

The luciferase activity of estrogen signaling activity was performed using the Dual-Luciferase Reporter kit (Promega, Germany). The ERE luciferase reporter was transfected together with the Renilla plasmid into the cells. Luciferase activity was measured after 24 h.

### Protein stability assays

About 10^5^ MCF-7 cells were seeded into twenty-four well plates and transfected with 50 uM USP1 siRNA or siControl. After 48 h, cells were treated with 100 uM cycloheximide (C7698, Sigma) for indicated time points. Samples were subject to western blot for ERα degradation.

### Co-immunoprecipitation assay

Immunoprecipitation was performed as described in previous study 18. The MCF-7 total cell lysls were pre-cleared with rabbit IgG for 2 h and subsequently immunoprecipitated with ERα antibody (SC8005, Santa Cruz) over night, while rabbit IgG (Santa Cruz) was used as the negative control. The bounded protein was analyzed by Anti-USP1 (SAB1406575, Sigma). For the overexpression experiment, HEK293 cells were transfected with 5ug GFP-USP1 and ERα plasmid in 10 cm dish. Cell lysates were pre-cleared with IgG and subsequently incubate with GFP (Ab290, Abcam) antibody, while rabbit IgG was used as the negative control. The bound proteins were analyzed by western blotting.

### Immunofluorescence assay

MCF-7 cells were fixed with 4% paraformaldehyde in PBS for 10 min, permeabilized with 0.2% Triton X-100 for 5 min, and blocked by 5% BSA in PBS for 1 h. A rabbit Anti-USP1 (SAB1406575, Sigma) rabbit antibody and mouse anti-ERα monoclonal antibody (SC-56833) were used, followed by Alexa Flour 647 (Invitrogen) anti-rabbit antibody and FITC-conjugated anti-mouse antibodies (Jackson ImmunoResearch, West Grove, PA). As negative controls, the samples were incubated with the secondary antibodies without primary antibodies. Images were acquired under conditions fulfilling the Nyquist criterion using Nikon A+ laser scanning confocal system with a 60X oil NA1.4 objective and pinhole size of 1.0 Airy Unit. The acquired pictures were further processed and assembled using ImageJ.

### Poly-ubiquitination detection assay

To directly detect the enriched overall ubiquitinated or K48-linked ubiqutinated ERα from the cell extracts, HEK293 cells were transfected with 4 ug Ub or 4 ug K48 Ubi plasmid, 2 ug ERα together with 0.5 ug Flag-USP1 or Flag-vector. After 48 h, cells were treated with 10uM MG132 and then the total protein was extracted and pre-cleared with 20ul protein A (santa cruz, SC-2001) for 2 h. The supernatant was collected and immunoprecipitated by ERα antibody. Western blot with HA antibody was performed to detect K48 poly-ubiquitinated ERα.

### Statistics

Student's *t*-test, Pearson correlation coefficient, and Cox regression analysis were used for comparisons. A *P*-value of < 0.05 was considered to be significant.

## Results

### USP1 is required for ERα signaling activity, which is elevated in human breast cancer and relates to poor survival in ERα positive breast cancer patients

**Figure 1:**
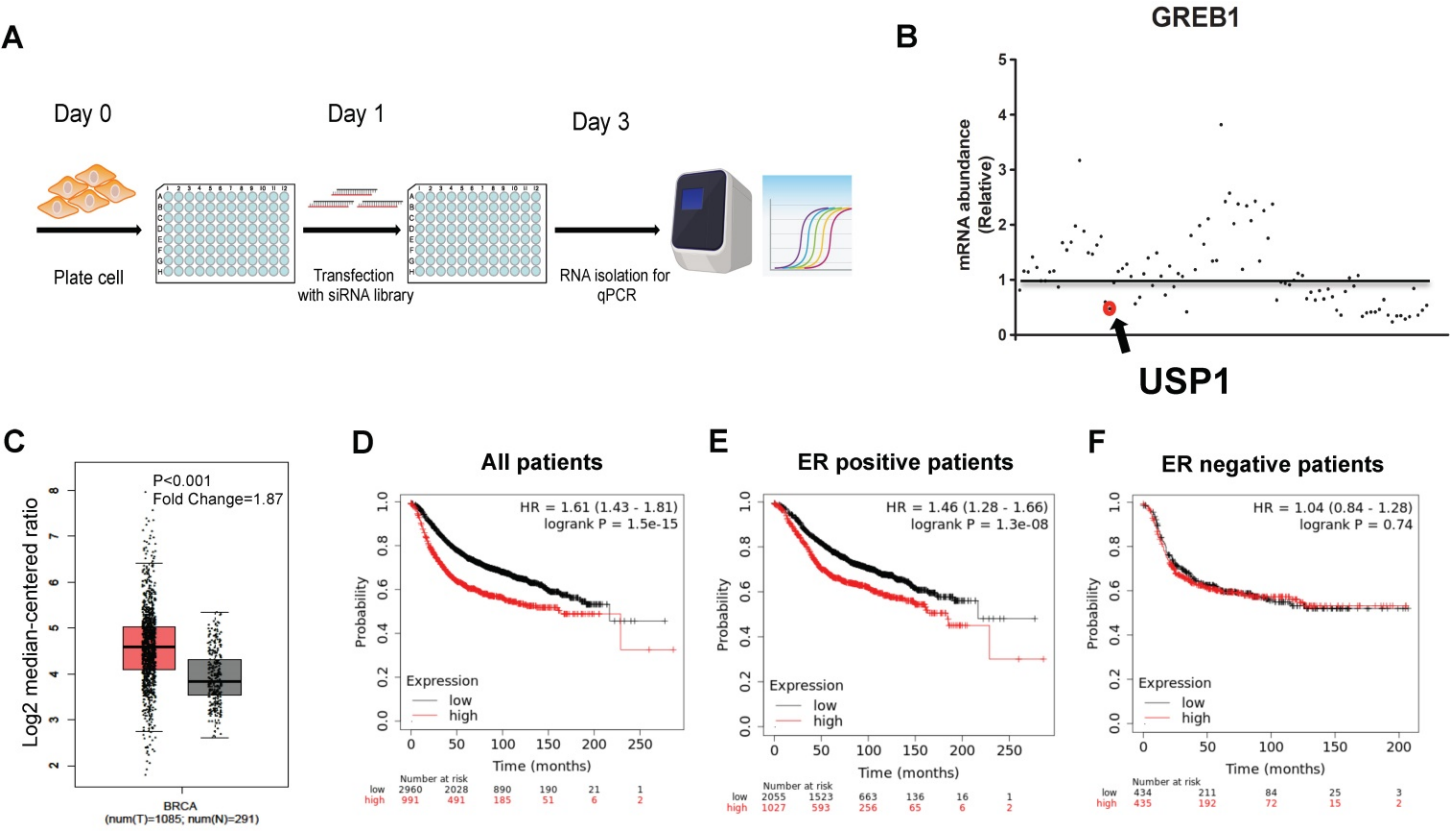
** USP1 is required for ERα signaling activity, which is elevated in human breast cancer and relates to poor survival in ERα positive breast cancer patients. A:** The procedure of siRNA screening for key DUBs for ERα signaling via DUBs siRNA library. MCF-7 cells were transfected with 20uM siRNA. After 48 hours, the whole genomic RNA was extracted from cells. Real-time PCR was utilized for quantitative gene expression analysis. The classical target gene GREB1 was used to indicate ERα signaling activity. **B:** The siRNA screening data showed that USP1 was required for GREB1 gene expression in MCF-7 cells. **C:** USP1 expression level was significantly elevated in breast cancer compared with normal breast tissue from TCGA database (https://www.genome.gov/Funded-Programs-Projects/Cancer-Genome-Atlas). **D:** USP1 expression was correlated with poor survival in human breast cancer from KMPLOT database (https://kmplot.com). **E:** USP1 expression was correlated with poor survival in ERα positive human breast cancer from KMPLOT database (https://kmplot.com). **F:** USP1 expression was correlated with poor survival in ERα negative human breast cancer from KMPLOT database (https://kmplot.com).

In order to identify the DUBs, which were required for ERα signaling in breast cancer, we utilized the DUBs siRNA library to silence each DUBs in MCF-7 cell. We used the classical ERα target gene to indicate the ERα signaling activity (Fig. 1A). The real-time PCR data showed that USP1 depletion dramatically inhibited GREB1 expression compared with siControl (Fold Change=0.48) (Fig. 1B). We further investigate the expression of USP1 in human breast cancer in public available datasets. The TCGA data showed that USP1 was elevated in human breast cancer compared with normal breast tissue (Fig. 1C). Besides, the expression of USP1 was correlated with poor survival in breast cancer (Fig. 1D). When we stratified the data, we found that the survival correlation only existed in ERα positive breast cancer group, but not in ERα negative breast cancer group (Fig. 1E-F).

### USP1 depletion inhibits ERα signaling activity in breast cancer

**Figure 2:**
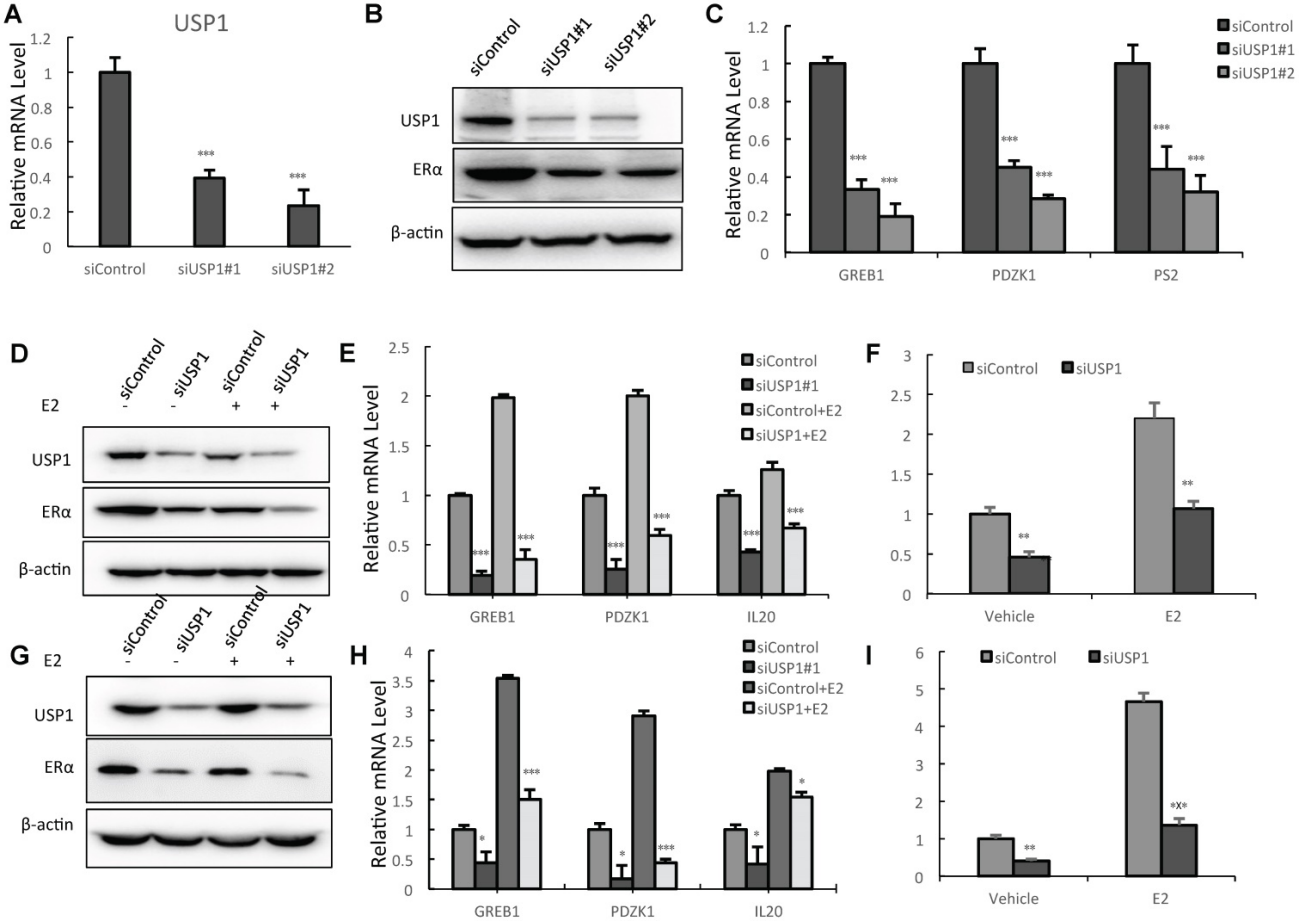
** USP1 depletion inhibits ERα signaling activity in breast cancer. A:** USP1 depletion effect by two independent siRNA oligos in MCF-7 cells. MCF-7 cells were transfected with siControl or siUSP1. After 48 hours, total RNA was extracted for gene expression analysis. *P<0.05; ** P<0.01; ***P<0.001 for target gene expression comparison. **B:** USP1 consumption decreased ERα protein levels in MCF-7 cells. MCF-7 cells were transfected with siControl or siUSP1. After 48 hours, cells were harvested for western blot analysis. USP1 and ERα protein levels were determined by Western blot. Actin was used as internal control. **C:** USP1 consumption decreased ERα target gene expression in MCF-7 cells. MCF-7 cells were transfected with siControl or siUSP1. After 48 hours, total RNA was extracted for gene expression analysis. *P<0.05; ** P<0.01; ***P<0.001 for target gene expression comparison. **D:** USP1 depletion decreases ERα protein levels in both vehicle and E2-treated conditions in MCF-7 cells. MCF-7 cells were transfected with siUSP1 or siControl. After 48 h, cells were treated with either ethanol or 10nM estradiol for 6 h. USP1 and ERα protein levels were determined by Western blot analysis. Actin was used as internal control. **E:** USP1 depletion decreases ERα target genes in both vehicle and E2-treated conditions in MCF-7 cells. MCF-7 cells were transfected with siUSP1 or siControl. After 48 h, cells were treated with either ethanol or 10nM estradiol for 6 h. Total RNA was prepared and the expression of the endogenous ERα target genes, IL20, GREB1, and PDZK1 were determined by qPCR. Shown are the results from three experiments. *P<0.05; ** P<0.01; ***P<0.001 for target gene expression comparison. **F:** USP1 depletion affects ERE-luciferase activity in MCF-7 cells. MCF-7 cells were transfected with siUSP1 or siControl together with ERE luciferase reporter plasmid. Cells were treated with 10 nM estradiol or vehicle. Luciferase activity was measured 48 h after transfection. Shown are the results from three experiments. *P<0.05; ** P<0.01; ***P<0.001 for luciferase activity comparison. **G:** USP1 depletion decreases ERα protein levels in both vehicle and E2-treated conditions in T47D cells. T47D cells were transfected with siUSP1 or siControl. After 48 h, cells were treated with either ethanol or 10nM estradiol for 6 h. USP1 and ERα protein levels were determined by Western blot analysis. Actin was used as internal control. **H:** USP1 depletion decreases ERα target genes in both vehicle and E2-treated conditions in T47D cells. T47D cells were transfected with siUSP1 or siControl. After 48 h, cells were treated with either ethanol or 10nM estradiol for 6 h. Total RNA was prepared and the expression of the endogenous ERα target genes, IL20, GREB1, and PDZK1 were determined by qPCR. Shown are the results from three experiments. *P<0.05; ** P<0.01; ***P<0.001 for target gene expression comparison. **I:** USP1 depletion affects ERE-luciferase activity in T47D cells. T47D cells were transfected with siUSP1 or siControl together with ERE luciferase reporter plasmid. Cells were treated with 10 nM estradiol or vehicle. Luciferase activity was measured 48 h after transfection. Shown are the results from three experiments. *P<0.05; ** P<0.01; ***P<0.001 for luciferase activity comparison.

We utilized two independent siRNAs to carry out the experiments. The real-time PCR data showed that USP1 depletion significantly decreases its mRNA level (Fig. 2A). The western blot data showed that USP1 depletion decreased ERα protein levels (Fig. 2B). The QPCR assay showed that USP1 depletion decreased the expression of ERα target genes, including GREB1 and PS2 (Fig. 2C). We further test USP1 effect on ERα signaling in both vehicle and E2-treated conditions. USP1 depletion could decrease ERα protein level in vehicle and E2-treated conditions in both MCF-7 and T47D cells (Fig. 2D and 2G). Consistently, USP1 depletion could dramatically decrease ERα target gene expression in MCF-7 and T47D cells, including IL20, GREB1, PS2 and PDZK1 (Fig. 2E and 2H; Supplementary Fig. 1A and 1B). In order to determine if USP1 knockdown could affect ERα transcriptional activity, we measure estrogen response element (ERE) luciferase activity in both MCF-7 and T47D cells. The luciferase assay shows that USP1 depletion decreases ERE luciferase activity in both MCF-7 and T47D cells (Fig. 2F and 2I).

### USP1 depletion inhibits cell proliferation and invasion in breast cancer

**Figure 3:**
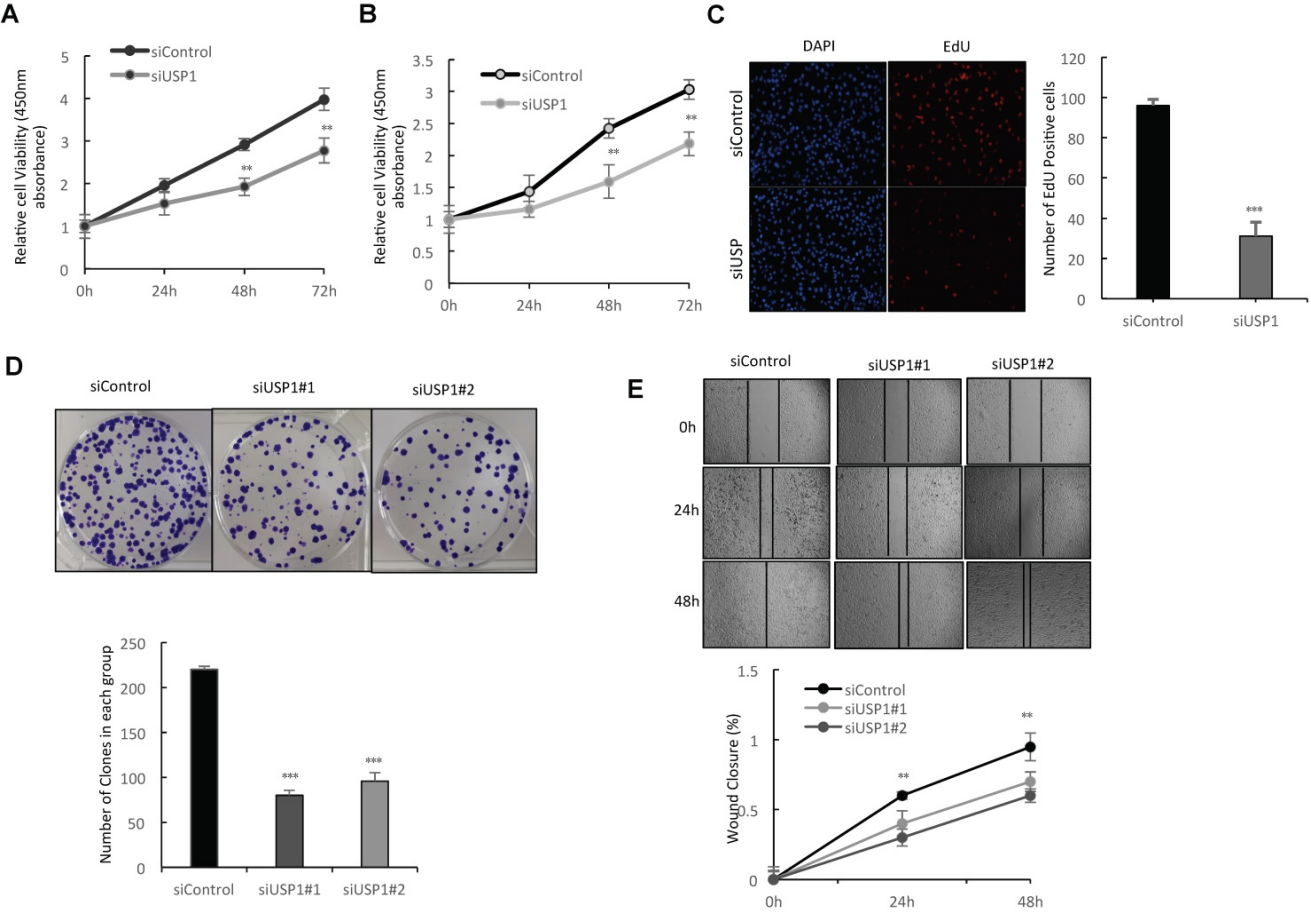
** USP1 depletion inhibits cell proliferation and invasion in breast cancer. A, B:** Depletion of USP1 inhibits the proliferation of breast cancer cells. MCF-7 and T47D were transfected with siControl or siUSP1. There were two different siRNA be used. After 24 hours, the assay of WST-1 was used to determine the cellar metabolic activity at indicated time points after infection. Experiments were done in triplicates. *P<0.05; ** P<0.01; ***P<0.001 for cell growth comparison. **C:** USP1 depletion inhibited the number of EdU positive breast cancer cells. MCF-7 cells were transfected with siControl or siUSP1. After 24 hours, EdU was added into the medium for 2 hours incubation. The absolute cell number was counted to indicate cell proliferation activity. **D:** Clone formation assay of MCF-7 cells transfected with indicated 50nM USP1 siRNA (mix of #1 and #2) or 50 nM control siRNA. Quantification of clone formation is shown at the indicated time points. Data are presented as ± SD. **, P<0.01, ***, P< 0.001 (student's t-test). **E:** Wound-healing assay of MCF-7 cells were transfected with siControl or siUSP1. Quantification of wound closure at the indicated time points. Data are presented as ± SD. **, P<0.01, ***, P< 0.001.

In order to investigate the impact of USP1 on breast cancer phenotypes, we deplete USP1 in breast cancer cells. WST assay shows that USP1 depletion significantly decreases breast cancer cell proliferation in MCF-7 and T47D cells (Fig. 3A-3B). Such phenotype was also confirmed by further EdU incorporation assay, while USP1 depletion significantly decreased the EdU positive cells in MCF-7 cells (Fig. 3C and Supplementary Fig. 1D). Clone formation assay shows that USP1 depletion dramatically inhibits the clone formation capacity in MCF-7 cells (Fig. 3D and Supplementary Fig. 1C). Besides, the wound-healing assay shows that USP1 knockdown decreases the wound closure speed in MCF-7 cells (Fig. 3E and Supplementary Fig. 1F).

### USP1 associates with ERα and regulates ERα stability

**Figure 4:**
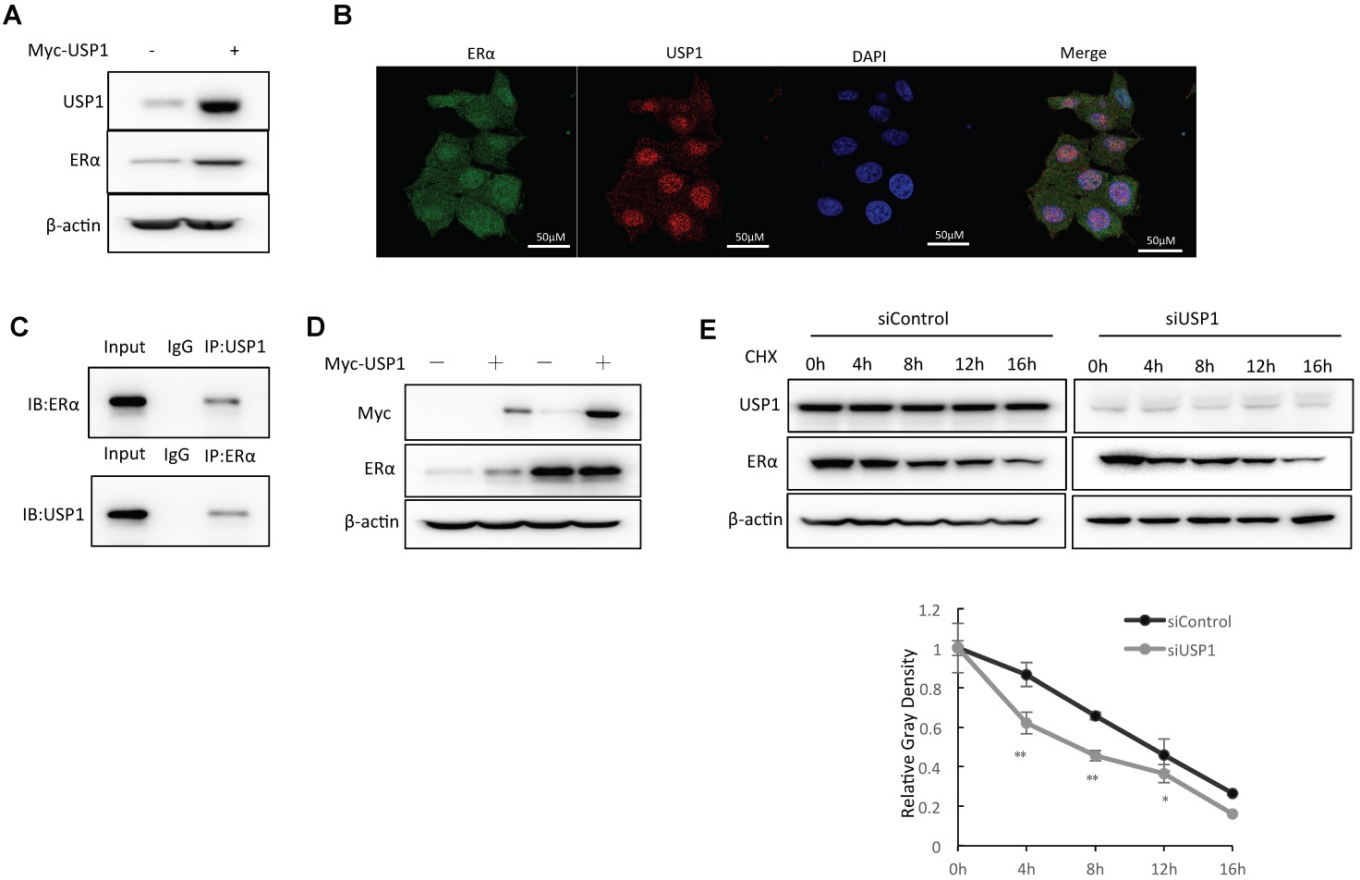
** USP1 associates with ERα and regulates ERα stability. A:** USP1 over-expression increased ERα protein level in MCF-7 cells. MCF-7 cells were transfected with 1ug Myc-USP1 plasmids or 1 ug Myc-vector. After 48 hours, cells were harvested for WB analysis. USP1 and ERα protein levels were determined by Western blot analysis. Actin was used as internal control. **B:** Intracellular localization analysis of USP1 and ERα by immunofluorescence assay. MCF7 cells were cultured in normal medium before fixation. Intracellular localization of USP1 (red) and ERα (green) were shown. Nuclei (blue) were stained with 4',6-diamidino-2-phenylindole (DAPI). **C:** Co-IP assay reveals association between endogenous USP1 and ERα in MCF7 cells. MCF-7 cells were harvested with RIPA lysis buffer. CO-IP was performed using antibody as indicated. **D:** In the presence of the proteasome inhibitor MG132, the stabilization effect of USP1 on ERα did not further increase ERα protein levels. HEK293 cells were transfected with 2 µg ERα plasmid and 0.5 µg Myc-tag or Myc-USP1 plasmids. After 24 h, cells were treated with 10 uM MG132/vehicle for 6 h. Cell lysates were prepared for Western blot analysis. The results are representative for three independent experiments. **E:** USP1 increases ERα half-life in MCF-7 cells. MCF-7 cells were transfected with 50uM siUSP1 siRNA or siControl. After 24 h, cells were treated with 100 µM cycloheximide/vehicle for indicated times. Cell lysates were prepared for Western blot analysis. The results are representative for three independent experiments. The ERα relative density was measured by Image J software.

We further overexpressed USP1 in MCF-7 cells and the WB data showed that USP1 could promote ERα protein level (Fig. 4A). The immuno-staining assay showed that both USP1 and ERα were located in the nuclear (Fig. 4B). The endogenous immuno-precipitation showed that USP1 could interact with ERα in MCF-7 cells (Fig. 4C). Since USP1 could associate with ERα in breast cancer cells, we further investigate the biological effect of such interaction. Since ERα could regulate its own expression, making it difficult to distinguish direct effects of USP1 on ERα mRNA or protein levels in the cell line. We utilize HEKC293 cells to investigate the mechanism. Co-transfection of ERα and USP1 in HEK293 cells shows that USP1 could increase ERα protein level, which effect could be minimized with the presence of the proteasome inhibitor MG132 (Fig. 4D). The protein half-life assay shows that USP1 could increase the protein stability of ERα (Fig. 4E).

### USP1 stabilizes ERα via inhibiting ERα K48-linked poly-ubiquitination

**Figure 5:**
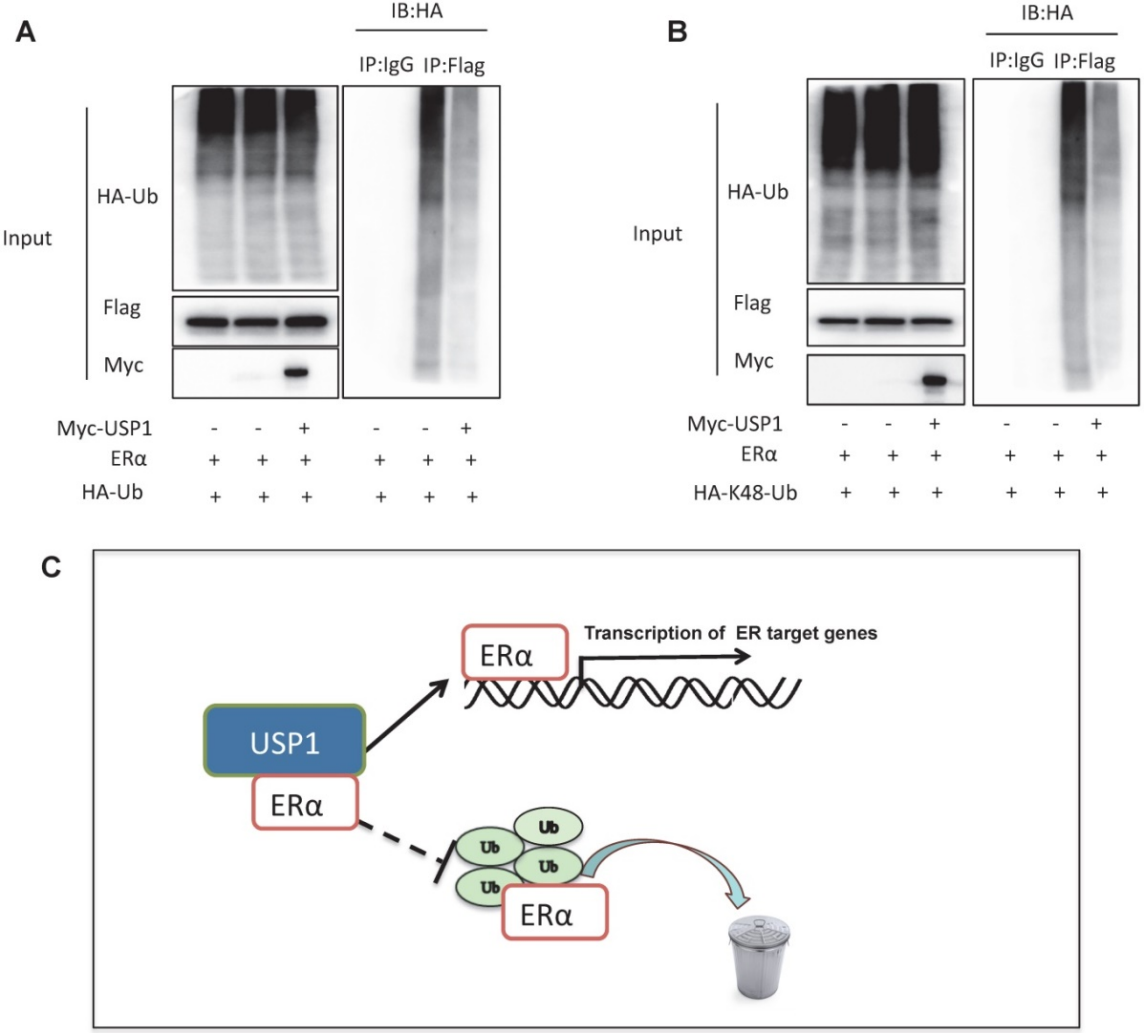
** USP1 stabilizes ERα via inhibiting ERα K48-linked poly-ubiquitination. A:** USP1 decreases poly-ubiquitination of ERα. HEK293 cells were transfected with 2 µg ERα plasmid, 0.5 µg HA Ub plasmid and 0.5 µg Myc-tag or Myc-USP1 plasmids. The cell extracts were immunoprecipitated with HA antibody. The poly-ubiquitinated ERα was detected via western blotting analysis. **B:** USP1 decreases K48-linked poly-ubiquitination of ERα. HEK293 cells were transfected with 2 µg ERα plasmid, 0.5 µg HA-K48 Ubi plasmid and 0.5 µg Myc-tag or Myc-USP1 plasmids. The cell extracts were immunoprecipitated with HA antibody. The K48 specific poly-ubiquitinated ERα was detected via western blotting analysis. **C:** USP1 protein is related to ERα, which promotes ERα target gene transcription by promoting ERα stability and inhibits ERα degradation by prohibiting ERα K48-linked polyubiquitination.

Since USP1 is one Deubiquitinating enzyme, we further investigated the role of USP1 in ERα poly-ubiquitination. The ubiquitination-based immuno-precipitation shows that USP1 could inhibit ERα overall poly-ubiquitination (Fig. 5A). Since K48-linked ubiquitination is the most common degradation manner, we examine the USP1 effect on K48-linked ubiquitination of ERα, which implicates that USP1 could inhibit K48-linked ubiquitination of ERα (Fig. 5B).

## Discussion

In our study, we identify a novel hit from DUBs siRNA genomic screening. We identify that USP1, which are elevated in human breast cancer samples and related to poor survival in ERα positive breast cancer patients. Besides, USP1 depletion inhibited ERα signaling activity and estrogen-stimulated cell proliferation and invasion. The mechanistic experiments revealed that USP1 associated with ERα and increased ERα stability via prohibiting ERα K48-linked poly-ubiquitination in breast cancer cells (Fig. 5C).

ERα belongs to the superfamily of nuclear receptor, which is encoded by ESR1 gene 19. The ERα protein consists of four family members, including one DNA binding domain, one ligand binding domain and two transcriptional activation domains 20. When ERα is activated by estrogen, it shuttles from the cytosol into the nuclear, which subsequently binds to the estrogen response element in the DNA and activates ERα target gene expression 6, 21. ERα is elevated in breast tumors and becomes the major driver for ERα positive cancer types 22. Based on the importance of ERα, targeting ERα signaling could be an effective strategy. The selective estrogen receptor modulators, such as tamoxifen, are the first line therapy for ERα positive breast cancer patients 21. However, most of the endocrine therapy patients will develop drug resistance, while the molecular mechanisms are not totally clear. Surprisingly, most of endocrine resistance breast tumors sill maintain ERα expression, which indicates the possibility that ERα also plays important roles in endocrine resistance 23. Base on this, modulating ERα protein expression and stability could be a plausible way for breast cancer therapeutics and endocrine resistance.

The protein ubiquitination process is counterbalanced by deubiquitination enzymes, which remove the ubiquitin chains from the target proteins. Currently, there are approximately 100 DUBs, while the USPs are the largest groups 24. Even several E3 ubiquitin ligases were reported to regulate ERα signaling in breast cancer, the process that how DUBs counteract with E3 ligases and facilitate ERα signaling is still not clear. We performed the DUBs siRNA screening for key deubiquitinases that controlled ERα signaling and identified USP1 as one of the major player. The USP1 gene was firstly identified in 1998, which protein is composed of 785 amino acids 25. The catalytic domain is located in the C-terminal of USP1 protein. Several studies confirmed that USP1 modulated DNA repair process via stabilizing a few DNA binding proteins 26. USP1 had low prevalence in gene mutation, but was elevated in several human cancers 15. In breast malignancy, USP1 was shown to promote triple negative breast cancer progression, but its function in ERα positive type is not clear 27. Our study showed that USP1 stabilized ERα via inhibiting K48-linked poly-ubiquitination of ERα, which provided a novel insight of DUBs in modulating hormone signaling and breast cancer progression.

In conclusion, we identified an interesting deubiquitinase USP1 in facilitating ERα signaling in breast cancer cells. USP1 could promote breast cancer cell invasion and proliferation via stabilizing ERα protein. As a novel modulator of ERα signaling, disturbing USP1 activity or affecting USP1 expression could be a plausible way to treat luminal types of breast cancer.

## Supplementary Material

Supplementary figures and tables.

## Abbreviations

ERα: Estrogen receptor α
USP1: ubiquitin specific peptidase
AF1 domain: activator function-1 domain
AF2 domain: activator function-2 domain
DBD domain: DNA binding domain
PR: progesterone receptor
HER2: human growth factor receptor-2
ERE: estrogen-response elements
ATCC: American type culture collection
STR: short tandem repeat
IP: immuno-precipitation
