# Supplementary material for: The deubiquitinating enzyme USP1 modulates ERα and modulates breast cancer progression
Source: J Cancer. 2020 Oct 12;11(23):6992–7000. doi: 10.7150/jca.50477 (PMC7591989; doi:10.7150/jca.50477)

Supplementary Figure Legends:

Figure 1A and B: USP1 consumption decreased PS2 expression in MCF-7 and T47D cells. MCF-7 cells were transfected with siControl or siUSP1. After 48 hours, total RNA was extracted for gene expression analysis. *P<0.05; ** P<0.01; ***P<0.001 for target gene expression comparison.

Figure 1C: Clone formation assay of T47D cells transfected with indicated 50nM USP1 siRNA (mix of #1 and #2) or 50nM control siRNA. Quantification of clone formation is shown at the indicated time points. Data are presented as ± SD. **, P<0.01, ***, P< 0.001 (student's t-test).

Figure 1D: USP1 depletion inhibited the number of EdU positive breast cancer cells. T47D cells were transfected with siControl or siUSP1. After 24 hours, EdU was added into the medium for 2 hours incubation. The absolute cell number was counted to indicate cell proliferation activity.

Figure 1E: Wound-healing assay of T47D cells were transfected with siControl or siUSP1. Quantification of wound closure at the indicated time points. Data are presented as ± SD. **, P<0.01, ***, P< 0.001.

Supplementary Figure 1

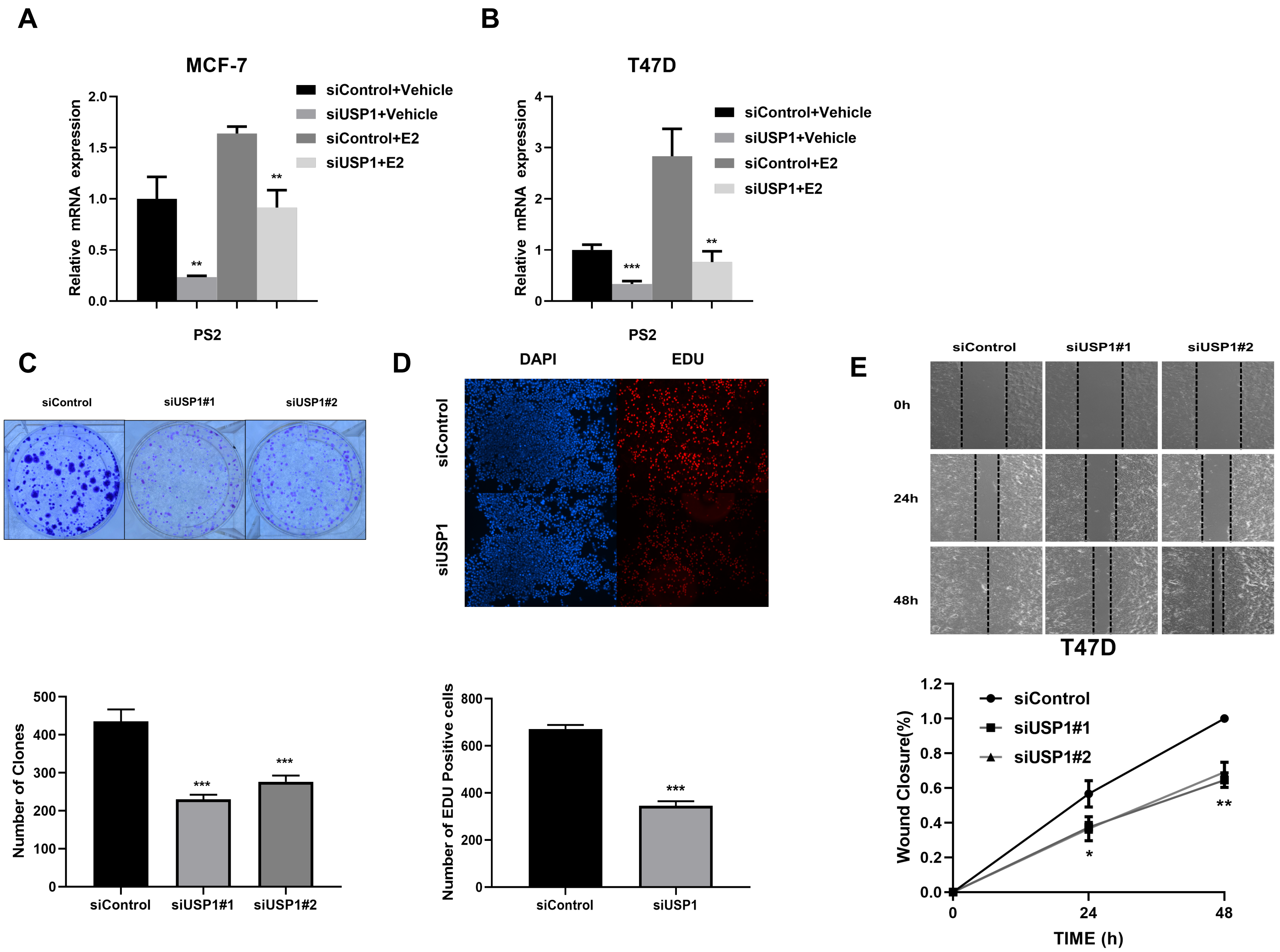

Supplement: Supplementary file 1 — Supplementary figures and tables. [file jcav11p6992s1.pdf]
